# Shining a Spotlight on Stigma: Exploring Its Impact on Oral Health-Seeking Behaviours Through the Lenses of Patients and Caregivers

**DOI:** 10.7759/cureus.63025

**Published:** 2024-06-24

**Authors:** Malik Zain Ul Abideen, Nashwa Alzaki Ali Bushara, Muhammad Nadeem Baig, Yasir Dilshad Siddiqui, Iqra Ejaz, Jawad Tareen, Ammar Ahmed Siddiqui

**Affiliations:** 1 Department of Dental Education and Research, Bakhtawar Amin Medical and Dental College, Multan, PAK; 2 Department of Preventive Dental Sciences, College of Dentistry, University of Ha'il, Ha'il, SAU; 3 Department of Preventive Dentistry, College of Dentistry, Jouf University, Sakaka, SAU; 4 Department of Oral Biology, Bakhtawar Amin Medical and Dental College, Multan, PAK; 5 Department of Medical Education, Bakhtawar Amin Medical and Dental College, Multan, PAK

**Keywords:** behaviours, perceptions, oral health, health equity, dental stigma

## Abstract

Introduction

The unique nature of a lack of good oral health, coupled with the lack of discussion and recognition surrounding the associated stigma, highlights it as a distinct issue. This stigma causes discomfort, devalues individuals, and necessitates urgent care and intervention. In Pakistan, a variety of reasons, including cultural beliefs, socioeconomic gaps, and poor access to dental care services, tend to exacerbate the stigma that is associated with dental care. This study aimed to determine the impact of stigma on oral health-seeking behaviours amongst the population of South Punjab in Pakistan.

Methodology

The study employed a qualitative design with a phenomenological approach, and the data collection was preceded by the administration of semi-structured interview guides and discussion guides to the patients and the consultant group, respectively. The targeted population was composed of patients who had reported stigmatization and a focus group of 10 dental consultants from various specialisations in dentistry. Data was collected until saturation from 16 patients who were recruited through the purposive convenience sampling technique.

Results

The study identified three themes, including perceived stigma impact on health-seeking behaviours and coping mechanisms; 10 subthemes emerged. Results showed social attitudes and unhealthy perceptions of oral conditions that lead to shame, loss of self-esteem, and lack of confidence among affected individuals. Behavioural reactions like mockery and discrimination further made it difficult for the participants who sought dental care and treatment. The study highlighted that stigma resulted in the avoidance of dental care, deterioration of oral health, and a tendency towards self-medication. People also used coping strategies such as hiding dental issues by avoiding social gatherings and seeking help from close friends to manage oral health stigma. Dental consultants had the strong opinion that care quality could be enhanced by utilising compassionate communication and patient education campaigns.

Conclusions

The experiences of patients and consultants related to dental stigma highlighted the complex interplay of sociocultural norms and healthcare practices. The study demonstrated that perceived stigma impacted the health-seeking behaviours of patients. Social support and education about oral health helped patients overcome this stigma. The study revealed that patients avoided dental treatments due to stigmatised behaviour from health professionals, a lack of affordability, and a feeling of shame to show and discuss the condition of their teeth, which got even worse due to self-remedy. The experiences of patients and consultants highlighted the need for increased advocacy, educational campaigns, and policies that can reduce inequalities in oral health and improve health equity. The study recommends specific intervention strategies and policy formulation to address oral health inequalities and contribute to advancing oral health equity in Pakistan.

## Introduction

Stigma, first described in the late 19th century by American sociologist Erving Goffman, refers to the societal tendency to evaluate and penalise behaviours perceived as deviant, including those related to health conditions like oral health. This marginalisation can lead to exploitation, avoidance, and social rejection, creating barriers to equitable access to healthcare services [1-3]. Unlike conditions such as HIV or mental health disorders, which can be hidden, oral health conditions are often visible and thus expose individuals to social judgement and stigma, impacting their self-esteem and overall well-being [4].

The phrase "the face is the window to the soul" emphasises how facial appearances often shape people's initial perceptions and behaviours towards others. Given its prominent visibility, it can be quite challenging to conceal one's oral health condition during social interactions. This is in contrast to other conditions that are often stigmatised in society, like HIV infection or mental health disorders, which are often hidden unless they are openly recognized. HIV stigma is a result of negative attitudes and beliefs towards people living with HIV [5,6].

The unique nature of a lack of good oral health, coupled with the lack of discussion and recognition surrounding the associated stigma, highlights it as a distinct issue. The stigma causes discomfort, devalues individuals, and necessitates urgent care and intervention [7].

Stigmatisation involves and leads to different ideologies, such as racism, ableism, and ageism. These beliefs can contribute to bias. Discrimination is a result of stigma, which leads to unfair treatment and societal conditions that restrict opportunities, resources, and overall well-being for individuals and institutions [8].

The way people view oral health and disease, their thoughts on the moral implications of having poor oral health (like neglecting oneself and being seen as socially inferior), and the cultural practices they follow (like brushing their teeth regularly and being concerned about hygiene) all play a role in the stigma surrounding oral health. Individuals with straight, white teeth are often associated with good health and prosperity, reflecting the ideals of the elite or dominant class [9]. Deviation from social norms in oral health has been associated with factors such as lower intelligence, advanced age, poverty, and illness. Individuals employ various methods to safeguard themselves against oral health issues that deviate from the norm. Because oral health is deeply intertwined with our social identities, aspirations for tooth care emerge as we plan to restore, cleanse, and reclaim our lives [10]. Oral health attributes that are not easily seen, such as biomedical aspects, are often overlooked in comparison to oral health attributes that are more visible and socially focused. Although cancer, diabetes, and depression can impact individuals from all walks of life, poor oral health is often linked to lower socioeconomic status and contributes to social inequality [11].

Feelings of shame, regret, and a sense of not being good enough can prevent individuals from seeking dental treatment [12]. For healthcare to significantly advance, that is, for better diagnosis, treatment, and health outcomes to take place, healthcare professionals need to treat all humans with equality and compassion. For instance, healthcare professionals can hold subconscious biases, which in turn might result in interpersonal situations that include micro-aggressions [13].

In Pakistan, a variety of reasons, including cultural beliefs, socioeconomic gaps, and poor access to dental care services, tend to exacerbate the stigma that is associated with dental care [14]. Cultural norms, ignorance, and financial problems are the reasons why those who are from the countryside or lower social classes suffer more from poor dental care. Oral health inequalities are still prevalent among populations with low incomes, and they are disproportionately impacted by avoidable oral diseases and dental issues [15].

Considering the context, it is imperative to focus on the impact of stigma on oral health-seeking behaviours in Pakistan. The overall aim of this research is to enable targeted interventions and policies to address the inequalities in dental treatments in Pakistan through multifaceted analyses of the connections between dental stigmas and oral healthcare-seeking behaviour.

## Materials and methods

This was a qualitative study with a phenomenological approach that included patients and dental consultants as participants. The target population was individuals in South Punjab, Pakistan, affected by oral health-related stigma, specifically patients with a history of such stigma and dental consultants who encounter such patients during consultations at tertiary care hospitals and were recruited during the consultation process.

Convenience purposive non-probability sampling was employed in this study to avoid the overrepresentation of a single variable. The sampling process continued until data saturation was achieved, which indicated new ideas or themes reported from interviews, and focus group discussions were exhausted. 

An information sheet outlining the objective, methodology, and proposed outcomes was shared with the participants, and written informed consent was obtained in the native language (Urdu) before conducting the interviews. The timing of the semi-structured interviews ranged from 19 to 33 minutes to allow for the collection of adequate information. In conducting interviews, face-to-face sessions were preferred because most of the participants agreed to participate in this type of interaction. Consent was obtained from the participants to record these interviews in audio format. These field notes were also taken to record vital contextual features such as the facial gestures, emotions, and minute changes in the reactions of the participants.

The interview guide and focus group discussion guide were prepared from an extensive literature search based on the objectives of the research and further refined using expert validation and pilot testing of the content for clarity, relevance, and cultural appropriateness. Interview and discussion guides were revised and modified based on expert feedback and pilot testing. The interview guide was translated into the local language (Urdu) by a bilingual expert and then translated back by another expert to ensure credibility.

Ethical clearance was obtained from the Institutional Review Board of Bakhtawar Amin Dental College and Hospital, Multan, Pakistan, with reference number COD 341/23.

The data was acquired through semi-structured interviews and focus group discussions via interview and discussion guides, respectively, from November 2023 to March 2024. Patients underwent interviews, and focus group sessions were held with the consultants. A total of 16 interviews were conducted with the patients, and 10 consultants from different oral health specialities participated in focus group sessions. 

Data collection and analysis were carried out simultaneously in this study, following a six-step process of Braun and Clarke's framework for thematic analysis that includes data collection, transcription of field notes and audiotapes, and iterative analysis [16].

The final transcripts were shared with the respective participants for member checking and approval via a national courier service of the country [17].

**Figure 1:**
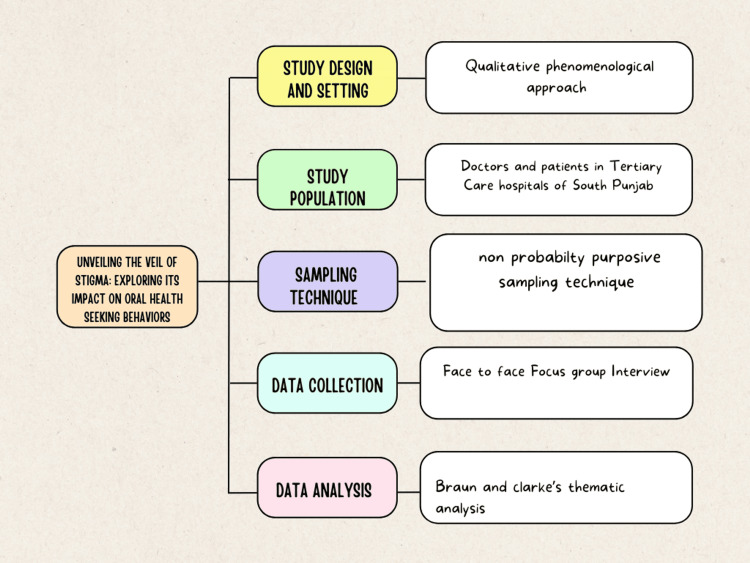
Methodology flowchart Image Credit: Author Malik Zain Ul Abideen with media element by Freya Saad via canva.com [18]

The data was coded manually based on thematic areas and descriptions that emerged during the data analysis process. The coding type employed in the analysis was primarily inductive, allowing themes and patterns to emerge directly from the data, supplemented by open coding techniques to ensure thorough exploration of the dataset. Tables were utilised to manage the large volume of the gathered information. Two researchers meticulously transcribed the audio tape recordings and field notes into text data, which was then counter-checked by the principal investigator to ensure quality. Similar codes were grouped to form major ideas or themes within the database. The analysis continued until the point of saturation (Figure 1).

## Results

The demographic characteristics of the study participants included 16 patients and 10 dental consultants. Among the patients, the gender distribution was equal, with eight males (50%) and eight females (50%). The age range varied, with five participants (31.25%) aged 18-25, six participants (37.5%) aged 26-35, three participants (18.75%) aged 36-45, and two participants (12.5%) aged 46-55. Socio-economic status among the patients showed a predominance of low socio-economic status (nine participants, 56.25%), followed by middle class (five participants, 31.25%), and high socio-economic status (two participants, 12.5%) (Table 1).

**Table 1:** Demographic characteristics of participants N/A: not applicable

Characteristics	Patients (n=16)	Consultants (n=10)
Gender		
Male	8	6
Female	8	4
Age range (years)		
18-25	5	0
26-35	6	3
36-45	3	5
46-55	2	2
Socioeconomic status		
Low	9	N/A
Middle	5	N/A
High	2	N/A
Speciality (consultants)		
Prosthodontics	N/A	2
Orthodontics	N/A	3
Periodontics	N/A	1
Oral and maxillofacial surgery	N/A	2
Endodontics	N/A	2
Years of experience (consultants)		
1-5 years	N/A	3
6-10 years	N/A	4
11-15 years	N/A	2
16+ years	N/A	1

The dental consultants comprised six males (60%) and four females (40%), with an age distribution of three consultants (30%) aged 26-35 years, five consultants (50%) aged 36-45 years, and two consultants (20%) aged 46-55 years. The specialities represented among the consultants included prosthodontics (two consultants, 20%), orthodontics (three consultants, 30%), periodontics (one consultant, 10%), oral and maxillofacial surgery (two consultants, 20%), and endodontics (two consultants, 20%). Regarding years of experience, three consultants (30%) had 1-5 years, four consultants (40%) had 6-10 years, two consultants (20%) had 11-15 years, and one consultant (10%) had over 16 years of experience (Table 1).

Thematic analysis of patient interviews revealed three main themes and 10 subthemes related to dental stigma. Patients internalised negative societal beliefs about oral health, leading to embarrassment and feelings of inferiority. Emotional distress, including depression and anxiety, significantly affected their mental well-being. Stigmatising behaviours like mockery and discrimination deterred timely dental care-seeking. Patients with systemic disorders faced additional challenges such as social isolation and discrimination from both the public and dental professionals (Table 2).

**Table 2:** Patients' experiences of dental stigma

Themes	Sub-themes	Representative quotations
Perceived stigma	Internalization of negative beliefs	Participants stressed that they had a lot of negative thoughts about their oral health issues as they internalized the societal norms and expectations and hence felt embarrassed or inferior as they conformed to them. Participant two mentioned that she felt ashamed "due to the condition of her present oral health, bad breath, and being afraid of being judged by other people and health care providers that I am much inferior to those with perfect smiles".
	Emotional distress	Most participants described negative emotions such as depression, anxiety, and low self-esteem as common experiences caused by the stigma imposed on their oral status. This distress translated into feelings of loneliness, patient depression, and less optimistic self-esteem as well. As participant seven voiced, "My oral health stigma makes me feel like I can never smile or laugh. I avoid social gatherings and events and give up my dream of becoming a standup comedian because I feel that my facial profile is a barrier to this."
	Stigmatizing behaviors from others	Participants enumerated prejudices they faced, like being mocked due to some visible dental anomalies. Indeed, such behaviour from friends and society in general made it hard to acquire dental care, resulting in avoidance and reluctance to seek dental care. Participant 11 said, "I have been the victim of discrimination and ridicule by other people since my dental problems are quite obvious. I even went to a tertiary care hospital for this reason, and the staff made fun of me. My oral hygiene routine has since been deemed bad and I have become reluctant to seek dental care."
	Reluctance due to any systemic disease/disorder	Participants faced isolation, avoidance, and disturbing comments on the basis of some systemic illnesses. Participant six stated, “I suffered from hepatitis C, and I faced harsh response from people and even healthcare providers when I went for scaling. During history when I told the dentist, their reaction changed immediately. They delayed my treatment by giving me multiple appointments, and I was waiting for hours. They avoided me and treated me with disrespect, I felt disrespected and degraded by these behaviours.”
Impact on health-seeking behaviors	Avoidance of dental care	The stigma of oral health disease was found to be the main reason to avoid dental care, as identified among participants. Many people mentioned being scared of or getting a judgmental look from the dental providers as the reason why they stretched the dental appointments to the minimum or avoided them completely. Participant five portrayed the stigma that prevented one from seeking regular dental check-ups and treatments. He asserted, "Due to my abnormal teeth and the delayed treatment, I always feared being stigmatised or judged by dental professionals, and thus I would avoid any dental check-up and treatment."
	Worsening of problems	During the interviews, participants shared that they were hiding their dental problems from others, but symptoms were getting out of hand and complications were developing steadily. It was really difficult to consult and get treatment because of the fear of being stigmatized or experiencing discrimination. The patients reported that they had postponed the treatment until their oral health issues became unbearable. Participating nine described, "I had to put off my dental needs for so long before I realized that the stigma led to the aggravation of my dental problems."
	Alternative remedies	The lack of financial resources made certain participants rely on either home remedies or self-medication and avoid visiting dental professionals altogether. This, too, made their oral health even worse. Participant 14 described the situation as follows: "I avoided visiting professional dental clinics due to a lack of funds and tried numerous home remedies and medications, and now I'm too embarrassed to visit the dentist as I damaged all my front teeth due to these home experiments."
Coping mechanisms	Covering up	The members mentioned meetings in which they hid their dental problems to avoid stereotypical rejection or unfavourable reactions from others. Most of the participants hid their smiles in social gatherings and kept themselves away from indulging in social interactions and meetings to cope with the stigma. Participant four confessed, “I avoided smiling during social interactions and was reluctant to smile openly at particular places that often made me awkward because I’m scared of other people's reactions."
	Seeking social support	The participants believed that having support from close people who understood and cared for them was an effective coping mechanism to overcome this dental stigma. Speaking to people who, essentially, understood their struggles and were ready to help and soothe the unwanted feeling of loneliness, gave them much-needed emotional support. As participant eight reported, "I discussed my feelings and dental problem with my friends and family, and they comforted me by understanding and listening to my feelings. My friend really helped me to overcome this and also rightly guided and motivated me to seek consultation and appropriate treatment with confidence."
	Advocacy and education	Some participants explained the role of education and awareness related to dental problems in overcoming stigma. They managed awareness campaigns within their communities to accept dental anomalies and seek support from dental professionals regarding oral health issues. Participant 12 stated, "I took an active role in advocating for the eradication of dental stigma in the community as well as advocating for equality and inclusion for people with oral problems. I also experienced stigma for a very long time, and the efforts and eagerness to put my oral health back on track through learning and awareness have given me the confidence to tell my story and influence positive change."

Stigma lowered patients' self-esteem, often causing them to avoid dental treatment. Many postponed appointments due to the fear of judgement, worsening their dental health. Financial constraints led some to use home remedies, worsening conditions. Patients hid dental problems to evade discrimination and awkward situations. Support from friends and family encouraged seeking treatment. Education and awareness about dental health were key coping strategies against stigma (Table 2).

Table 3 summarises the perspectives shared by dental consultants during focus group discussions regarding strategies to mitigate dental stigma. Consultants emphasised issues such as patient reluctance to disclose information due to stigma, resulting in compromised treatment outcomes. Strategies discussed included fostering empathetic patient interactions, enhancing patient education about oral health, and engaging in community outreach programmes to raise awareness. Consultants also highlighted the need for improved cultural competency training in dental education and recommended workshops to better equip dental professionals to manage stigma-related challenges (Table 3).

**Table 3:** Dental consultants' perspectives regarding dental stigma

Themes	Key findings
Impact of stigma on treatment quality	Stigma leads patients to conceal information crucial for effective treatment. One consultant stated, "Patients often fail to disclose information because of stigma, thus negating any improvement in treatment."
Anxiety and negative dental experiences	Anxiety stemming from perceived stigma adversely affects patient experiences. A consultant noted, "Stigmatized individuals feel anxious visiting dentists, making their dental experience negative."
Current strategies to reduce stigma	Empathetic listening and addressing patient concerns are crucial. One consultant emphasized, "I always try to be very open and friendly with my patients." Educating patients about oral health and treatment options also helps reduce shame.
Community engagement and awareness campaigns	Consultants highlighted the need for community awareness campaigns to demystify oral health issues and shield patients from discrimination. One consultant suggested, "Increased effort in community awareness programmes may assist in demystifying oral health problems."
Training and cultural competency	Concerns were raised about insufficient training in managing stigma during dental education. Consultants advocated for workshops and guidelines on managing stigma in dental practice. One mentioned, "Organizing workshops and formulating guidelines on managing stigma will make situations like these easier to deal with for all of us."

## Discussion

The research assessed the complex process of stigmatization regarding oral health-seeking behaviours and discussed vital sociocultural issues related to the interventions of dental consultants in the context of stigma. The research utilized a qualitative phenomenological approach to unfold the diverse lived experiences, attitudes, and ways to cope with this situation, which is essential for formulating policies to promote equal access to oral health services in the country.

The results demonstrated that stigma occupies a central role and affects people’s psychological states and their actions and behaviours towards oral health. A number of patients described instances where they felt stigma, such as shame, embarrassment, and discrimination, due to their dental appearance and oral health issues. The negative beliefs internalized by the patients when they faced oral health problems resulted in depression, anxiety, and low self-esteem, making the burden of the dental health problem heavier. The findings of the study were consistent with another study that demonstrated low self-esteem, depression, and psychological issues in patients with dental stigma [19]. It further weakened their desire to pay attention to their oral health and to seek necessary dental care, and it also resulted in lingering dental treatment, thereby worsening the oral health condition.

Furthermore, patients’ experiences emphasised that they encountered incidences of stigma from people and even dental professionals, which included teasing, mockery, and discrimination, discouraging them from seeking dental services. Discrimination by dentists was found to be another key factor that would deter people from seeking dental care. The threat of being judged or criticized by dental professionals during the consultation process led people to avoid such appointments. This pattern of avoidance behaviour negatively affected oral health and, at the same time, recreated the stigma by creating a societal perception that seeking dental professionals is not helpful. A study conducted in 2020 also highlighted that cultural competency training and the inclusion of social determinants of health are the primary issues that must be addressed through curriculum and policymaking [20]. 

In addition, the study revealed the negative effects of stigma on people with systemic illnesses, who, aside from being physically separated from others, received rejection and many other types of discrimination from the community as well as from health care providers. Such structural naïveté not only added to the patients’ oral health problems but also highlighted the structural causes of health disparities and stigma, which calls for multi-sectoral approaches to health. Oral health disparities can be addressed through the involvement of community leaders and non-governmental organisations through the basic principles of social responsibility and accountability of healthcare institutions [21].

There are various cultural attitudes and behaviours that patients employ to address and avoid the impacts of stigma. Most of the patients hid their dental issues from the public to avoid rejection and embarrassment; others sought help and advice from close friends or family members. These coping mechanisms highlighted the need for a social support system, participation in social activities, and challenging stigma by empowering marginalised populations through awareness, education, and inclusivity. By fostering a supportive environment, individuals can feel more comfortable seeking help and addressing their dental issues without fear of judgment [22]. 

Dental consultants also added perspectives regarding challenges in combating dental stigma. Clinicians emphasized the negative impact of stigma on the quality of care and patient-clinician relations, stressing the importance of consistent, non-judgmental, yet culturally sensitive training of dentists to decrease the effects of stigma. Consultants also provided insight that patient education, equity, and compassionate communication help to manage anxiety and fear related to stigma. A study conducted in 2020 highlighted the role of empathetic communication in managing patient anxiety and increasing trust in dental professionals [23].

Moreover, the results showed the ineffectiveness of consultants in tackling stigma-related problems due to a lack of preparedness and a lack of clear instructions or policies on managing such issues within dental colleges. Hence, it indicated a need for fundamental changes in dental curricula and practice, whereby dental professionals are adequately prepared to tackle stigma and provide equal and quality oral healthcare to all [24].

The study's findings underscored the necessity for targeted interventions and government involvement to combat stigma and ensure equitable access to dental services through financial support. It emphasised the importance of collaborative efforts involving dental professionals, community leaders, and government agencies to foster an inclusive culture that prioritises safety and equity in oral healthcare provision, irrespective of patients' socioeconomic backgrounds. Such initiatives are crucial for addressing systemic disparities and promoting equal access to dental care across diverse populations in Pakistan.

Despite the findings of this study, which provide insights into stigma related to oral health-seeking behaviours among the general population, some limitations of the study are as follows: Firstly, by approaching the topic qualitatively, the generalization of the results is limited to other populations and communities. Although researcher bias was removed through member checking, counter-checking from researchers, and coding, the subjective nature of the results might result in response bias, and it also limits the objective interpretation of the results. Therefore, future studies should be carried out to exclude these limitations through large-scale quantitative studies, cross-sectional studies among different regions, and comparative analyses to gain a better understanding of oral health stigma.

## Conclusions

The experiences of patients and consultants related to dental stigma highlighted the complex interplay of sociocultural norms and healthcare practices. The study demonstrated that perceived stigma impacted the health-seeking behaviours of patients. Social support and education about oral health helped patients overcome this stigma. The study revealed that patients avoided dental treatments due to stigmatised behaviour from health professionals, a lack of affordability, and a feeling of shame to show and discuss the condition of their teeth, which got even worse due to self-remedy. The experiences of patients and consultants highlighted the need for increased advocacy for specific interventions and policies that can reduce inequalities in oral health and improve health equity. The findings suggested that strategic planning from the governing bodies is required to make sure all stakeholders provide oral healthcare services while respecting the dignity of vulnerable people. This study concludes by recognising good oral health as a fundamental human right, without discrimination and inequality.

## Appendices

Appendix 1

Semi-Structured Interview Guide for Patients

Can you please introduce yourself (age, gender, and occupation)?

Can you please explain the oral health problems you have experienced?

How have these issues impacted your daily life and well-being?

If you are comfortable, please describe any instance in which you felt judged or discriminated against because of your oral health condition.

Can you share any specific experiences where you felt stigmatised due to your oral health?

How has the fear of stigma affected your decision to seek dental care?

Can you share if you avoided going to the dentist because of concerns about being judged or stigmatised?

How do you cope with the stigma related to your oral health issues?

What changes do you think could help reduce stigma related to oral health in your community?

Appendix 2

Focus Group Discussion Guide for Dental Consultants

Please, each of you briefly introduce yourself and your speciality.

How prevalent is stigma related to oral health among your patients?

Can you share any specific instances where you observed or felt patients feeling stigmatised?

How does stigma impact the quality of care you can provide to your patients?

Have you noticed any patterns or correlations among different demographics (age, gender, and socio-economic status) and dental stigma?

What strategies do you use to help reduce the stigma associated with oral health issues?

What additional measures or interventions do you believe could be implemented to reduce stigma and improve patient care?

How well-prepared do you feel dental professionals are to handle issues related to stigma?

What kind of training or resources would help you and your colleagues better address stigma in your practice?

Is there anything else you would like to add about the impact of stigma on oral health and patient care?
